# Variation in presepsin and thrombomodulin levels for predicting COVID-19 mortality

**DOI:** 10.1038/s41598-023-48633-0

**Published:** 2023-12-06

**Authors:** Azusa Yamazaki, Yoko Nukui, Takahiro Kameda, Ryoichi Saito, Yuki Koda, Naoya Ichimura, Shuji Tohda, Ryunosuke Ohkawa

**Affiliations:** 1 Department of Clinical Bioanalysis and Molecular Biology, Graduate School of Medical and Dental Sciences, Tokyo Medical and Dental University (TMDU), 1-5-45 Yushima, Bunkyo-ku, Tokyo, 113-8510 Japan; 2 Clinical Laboratory, Tokyo Medical and Dental University (TMDU) Hospital, 1-5-45 Yushima, Bunkyo-ku, Tokyo, 113-8510 Japan; 3 Department of Infection Control and Prevention, Tokyo Medical and Dental University (TMDU) Hospital, 1-5-45 Yushima, Bunkyo-ku, Tokyo, 113-8510 Japan; 4 Department of Infection Control and Laboratory Medicine, Kyoto Prefectural University of Medicine, 465 Kajii-cho, Kawaramachi-Hirokoji, Kamigyo-ku, Kyoto, 602-8566 Japan; 5 Department of Molecular Microbiology, Graduate School of Medical and Dental Sciences, Tokyo Medical and Dental University (TMDU), 1-5-45 Yushima, Bunkyo-ku, Tokyo, 113-8510 Japan

**Keywords:** SARS-CoV-2, Biomarkers, Prognostic markers

## Abstract

Coronavirus disease (COVID-19) has caused extensive mortality globally; therefore, biomarkers predicting the severity and prognosis of COVID-19 are essential. This study aimed to evaluate the application of presepsin (P-SEP) and thrombomodulin (TM), which are biomarkers of sepsis and endothelial dysfunction, respectively, in the prognosis of COVID-19. Serum P-SEP and TM levels from COVID-19 patients (n = 183) were measured. Disease severity was classified as mild, moderate I, moderate II, or severe based on hemoglobin oxygen saturation and the history of intensive care unit transfer or use of ventilation at admission. Patients in the severe group were further divided into survivors and non-survivors. P-SEP and TM levels were significantly higher in the severe group than those in the mild group, even after adjusting for creatinine values. In addition, TM levels were significantly higher in non-survivors than in survivors. Changes in the P-SEP levels at two time points with an interval of 4.1 ± 2.2 days were significantly different between the survivors and non-survivors. In conclusion, TM and continuous P-SEP measurements may be useful for predicting mortality in patients with COVID-19. Moreover, our data indicate that P-SEP and TM values after creatinine adjustment could be independent predictive markers, apart from renal function.

## Introduction

Coronavirus disease (COVID-19), caused by severe acute respiratory syndrome coronavirus 2 (SARS-CoV-2), is a novel infectious disease that originated in December 2019 in Wuhan city, Hubei Province, China, and developed into a pandemic in early 2020. Japan reported the first case of COVID-19 on January 16, 2020. To date, the total number of SARS-CoV-2-infected people has exceeded 500 million globally, and COVID-19 has caused six million fatalities. On average, half a million people per day were newly infected between May and June 2022. The global vaccination strategy against COVID-19 has prevented the infection, but the dynamics of SARS-CoV-2 variants could trigger a new wave at any time. COVID-19 is known to cause severe pneumonia, resulting in extensive morbidity and mortality globally. Specific therapies, such as extracorporeal membrane oxygenation therapy, are essential to reducing the mortality rate^1–3^. However, for reasons including the invasiveness of the therapy and limited availability of the equipment, appropriate treatment based on the patient’s condition is required. Therefore, various studies have been conducted to identify biomarkers for predicting the severity and mortality of COVID-19, where acute-phase proteins, coagulation markers, and endothelial dysfunction markers have been listed as potential candidates^4^. With regard to inflammation, the levels of acute-phase proteins, such as C-reactive protein (CRP) and interleukin-6 (IL-6), and white blood cells (WBC) can be elevated in patients with SARS-CoV-2 infection. In addition to the conventional inflammatory markers, serum presepsin (P-SEP), which is a sepsis marker, has also garnered attention in SARS-CoV-2 infection^5^.

P-SEP is a soluble cluster of differentiation (CD)14 subtype, which is produced intracellularly by enzymatic truncation of the CD14 N-terminal domain during bacterial phagocytosis by blood cells^6^. Since P-SEP is released into the bloodstream in response to bacterial infections, and the serum levels increase more specifically and earlier than other biomarkers, the quantification of serum P-SEP is useful for the early diagnosis of sepsis^6–9^. P-SEP levels also correlate with septic organ damage, and P-SEP increases in cases of acute respiratory distress syndrome (ARDS), with or without bacterial infection^10^. P-SEP levels do not increase as remarkably in viral infections as they do in bacterial infections; however, recent studies have presented various intriguing results wherein P-SEP levels were elevated even in patients with COVID-19, proving useful for predicting severity, particularly mortality^11–13^, respiratory distress^14–16^, and the duration of hospitalization^17^. However, P-SEP levels can fluctuate based on renal function^18,19^, and adjusting their levels using serum creatinine (CRE) levels might be necessary for the diagnosis of patients with renal failure^20^. Some patients with severe COVID-19 also show renal dysfunction, which might contribute to elevated P-SEP levels, although this point remains unaddressed in the literature.

Thrombomodulin (TM) is expressed in vascular endothelial cells and has been identified as a candidate biomarker for endothelial dysfunction; however, few studies have been reported to date^4^. SARS-CoV-2 infects alveolar epithelial cells via angiotensin-converting enzyme 2 receptors, which are also expressed in vascular endothelial cells^21^. Cell membrane disruption has been observed in cells infected with the virus, and the lungs of patients with COVID-19 show severe endothelial impairment^22^. Consistent with these reports, elevated serum and plasma TM levels have been observed in non-survivor patients with COVID-19^23,24^, and other studies have also reported poor outcomes^25–28^. Importantly, TM is also excreted by the kidney; thus, similar to P-SEP, kidney failure should be considered when evaluating TM levels. Hence, TM levels in the blood may be another useful biomarker, but the applicability remains controversial. Moreover, most studies have suggested the usefulness of P-SEP and TM levels using only a single measurement, and the changes in these levels have not been fully classified.

This study aimed to evaluate the serum P-SEP and TM level dynamics for the prognosis and prediction of COVID-19 severity after adjusting for CRE levels and comparing them with other COVID-19 biomarkers.

## Results

### Severity classification

**Figure 1:**
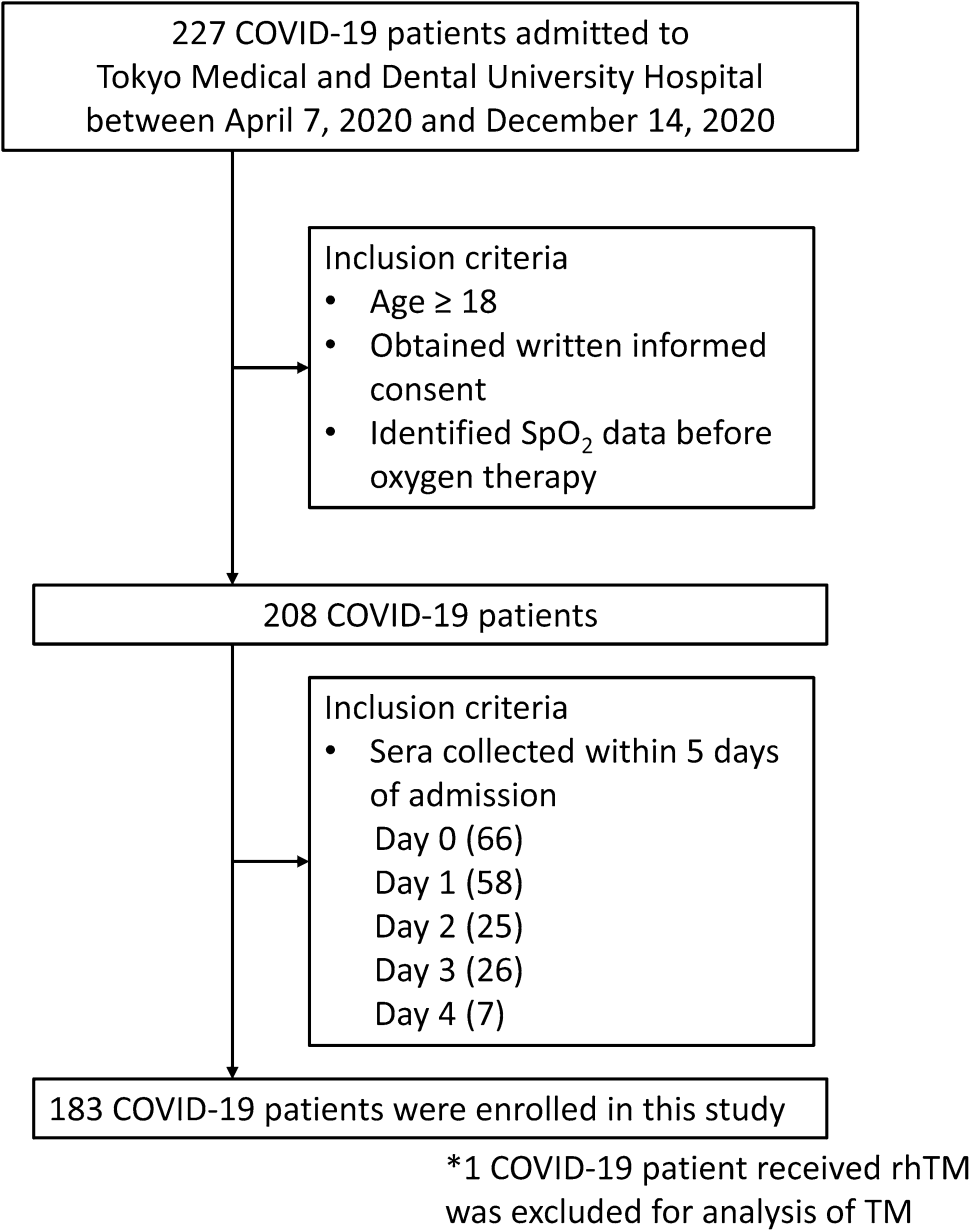
Patients’ selection flow chart. *COVID-19* coronavirus disease 2019, *SpO*_*2*_ oxygen saturation, *TM* thrombomodulin, *rhTM* recombinant human thrombomodulin.

We analyzed the stored sera from 183 patients within five days of admission according to the criteria in this study (Fig. 1). Disease severity was classified into four stages with reference to the Guidelines of the Diagnosis and Treatment of Novel Coronavirus issued by the Ministry of Health, Labour and Welfare, Japan, as follows^29^: mild (n = 104), moderate I (n = 24), and moderate II (n = 8) based on hemoglobin oxygen saturation (SpO_2_) values (≥ 96%, > 93 to < 96%, and ≤ 93%, respectively) which were obtained at admission before commencing oxygen therapy (Table 1). Moreover, 47 patients were classified as severe based on their history of intensive care unit (ICU) transfer or use of ventilation at admission to the Tokyo Medical and Dental University Hospital. Patients in the severe group were further divided into survivors (n = 34) and non-survivors (n = 13). In addition, among the patients in the severe group, data for those who underwent second P-SEP and TM measurement after admission with an interval of 4.1 ± 2.2 days between the two tests were extracted (survivors, n = 21–25, and non-survivors, n = 12–13).

**Table 1:** Demographics and laboratory characteristics among the severity groups.

	Total	Mild	Moderate I	Moderate II	Severe	*p*-value
		SpO_2_ ≥ 96%	93% < SpO_2_ < 96%	SpO_2_ ≤ 93%	ICU admission or ventilator support	
Age (mean ± SD)	56.3 ± 18.7	49.7 ± 18.8	62.0 ± 14.8	57.9 ± 11.4	67.7 ± 14.7	< 0.001*
Sex (male/female)	183 (123/60)	104 (63/41)	24 (16/8)	8 (5/3)	47 (39/8)	0.060
Death, n (%)	16 (8.7)	3 (2.9)	0 (0.0)	0 (0.0)	13 (27.7)	–
Length of hospital stay (days)	15.2 ± 16.8	11.5 ± 8.7	20.8 ± 29.7	18.4 ± 12.8	20.2 ± 20.0	0.010*
Laboratory data						
WBC, 10^3^/µL	6.4 ± 4.1 (n = 183)	5.3 ± 2.2 (n = 104)	6.2 ± 2.6 (n = 24)	5.6 ± 2.5 (n = 8)	9.2 ± 6.4 (n = 47)	< 0.001*
RBC, 10^4^/µL	451.6 ± 72.2 (n = 183)	463.4 ± 71.3 (n = 104)	464.5 ± 62.1 (n = 24)	491.5 ± 65.8 (n = 8)	412.0 ± 66.0 (n = 47)	< 0.001*
Hb, mg/dL	13.7 ± 2.0 (n = 183)	14.0 ± 2.0 (n = 104)	14.2 ± 1.9 (n = 24)	14.7 ± 1.6 (n = 8)	12.6 ± 2.0 (n = 47)	< 0.001*
Ht, %	40.2 ± 5.6 (n = 183)	41.2 ± 5.4 (n = 104)	41.3 ± 5.1 (n = 24)	42.3 ± 3.1 (n = 8)	37.0 ± 5.3 (n = 47)	< 0.001*
PLT, 10^4^/µL	22.9 ± 9.0 (n = 183)	22.6 ± 7.9 (n = 104)	22.7 ± 11.5 (n = 24)	20.8 ± 9.3 (n = 8)	24.1 ± 10.0 (n = 47)	0.663
PT, %	85.6 ± 17.1 (n = 182)	92.1 ± 14.7 (n = 103)	83.7 ± 11.9 (n = 24)	77.9 ± 17.8 (n = 8)	73.8 ± 17.5 (n = 47)	< 0.001*
PT-INR	1.1 ± 0.2 (n = 182)	1.1 ± 0.1 (n = 103)	1.1 ± 0.1 (n = 24)	1.2 ± 0.2 (n = 8)	1.2 ± 0.2 (n = 47)	< 0.001*
APTT, s	36.3 ± 14.3 (n = 182)	32.9 ± 5.3 (n = 103)	33.5 ± 3.9 (n = 24)	32.1 ± 5.5 (n = 8)	45.8 ± 24.6 (n = 47)	< 0.001*
APTT ratio	1.3 ± 0.5 (n = 182)	1.2 ± 0.2 (n = 103)	1.2 ± 0.1 (n = 24)	1.2 ± 0.2 (n = 8)	1.7 ± 0.9 (n = 47)	< 0.001*
Fbg, mg/dL	459.0 ± 137.8 (n = 164)	416.7 ± 136.9 (n = 94)	521.4 ± 148.1 (n = 17)	516.5 ± 123.0 (n = 8)	513.4 ± 106.9 (n = 45)	< 0.001*
D-dimer, µg/mL	2.5 ± 6.6 (n = 181)	1.4 ± 3.7 (n = 103)	1.6 ± 2.3 (n = 24)	9.4 ± 21.9 (n = 7)	4.6 ± 7.7 (n = 47)	< 0.001*
CRP, mg/dL	6.03 ± 7.47 (n = 183)	3.10 ± 5.44 (n = 104)	6.23 ± 3.90 (n = 24)	10.63 ± 9.59 (n = 8)	11.62 ± 8.84 (n = 47)	< 0.001*
LDH, U/L	312.7 ± 212.1 (n = 183)	236.3 ± 76.0 (n = 104)	315.0 ± 103.7 (n = 24)	406.1 ± 89.5 (n = 8)	464.6 ± 346.6 (n = 47)	< 0.001*
TP, g/dL	7.1 ± 0.8 (n = 137)	7.4 ± 0.7 (n = 81)	7.4 ± 0.6 (n = 15)	7.7 ± 0.8 (n = 8)	6.4 ± 0.7 (n = 33)	< 0.001*
Alb, g/dL	3.4 ± 0.8 (n = 181)	3.8 ± 0.7 (n = 102)	3.3 ± 0.6 (n = 24)	3.4 ± 0.7 (n = 8)	2.5 ± 0.5 (n = 47)	< 0.001*
UN, mg/dL	19.8 ± 15.6 (n = 183)	17.1 ± 15.3 (n = 104)	15.2 ± 5.6 (n = 24)	12.1 ± 4.4 (n = 8)	29.6 ± 17.1 (n = 47)	< 0.001*
CRE, mg/dL	1.1 ± 1.4 (n = 183)	1.1 ± 1.6 (n = 104)	0.8 ± 0.3 (n = 24)	0.7 ± 0.2 (n = 8)	1.2 ± 1.4 (n = 47)	0.070
eGFR, mL/min/1.73 m^2^	74.4 ± 30.5 (n = 183)	76.5 ± 28.7 (n = 104)	73.8 ± 20.3 (n = 24)	96.0 ± 37.8 (n = 8)	66.4 ± 35.4 (n = 47)	0.016*
UA, mg/dL	5.2 ± 2.2 (n = 163)	5.2 ± 2.0 (n = 100)	5.6 ± 2.4 (n = 22)	4.0 ± 0.8 (n = 8)	5.1 ± 2.7 (n = 33)	0.156
Ca, mg/dL	8.7 ± 0.6 (n = 163)	9.0 ± 0.6 (n = 90)	8.7 ± 0.6 (n = 21)	8.8 ± 0.4 (n = 8)	8.2 ± 0.5 (n = 44)	< 0.001*
IP, mg/dL	3.1 ± 0.8 (n = 146)	3.1 ± 0.7 (n = 82)	2.9 ± 0.6 (n = 19)	2.7 ± 0.3 (n = 7)	3.4 ± 1.2 (n = 38)	0.118
AST, U/L	41.5 ± 32.4 (n = 183)	32.5 ± 20.1 (n = 104)	40.7 ± 18.4 (n = 24)	59.0 ± 50.6 (n = 8)	58.9 ± 46.2 (n = 47)	< 0.001*
ALT, U/L	39.1 ± 35.2 (n = 183)	32.7 ± 30.2 (n = 104)	39.5 ± 24.2 (n = 24)	63.4 ± 71.6 (n = 8)	49.0 ± 38.5 (n = 47)	0.001*
γ-GTP, U/L	78.7 ± 78.8 (n = 180)	62.6 ± 62.3 (n = 101)	74.9 ± 63.4 (n = 24)	103.9 ± 94.0 (n = 8)	110.9 ± 103.2 (n = 47)	0.001*
T-Bil, mg/dL	0.7 ± 0.3 (n = 182)	0.7 ± 0.3 (n = 103)	0.7 ± 0.3 (n = 24)	1.0 ± 0.4 (n = 8)	0.6 ± 0.3 (n = 47)	0.026*
CK, U/L	259.5 ± 1046.6 (n = 180)	177.1 ± 861.5 (n = 101)	141.7 ± 254.9 (n = 24)	307.8 ± 472.0 (n = 8)	488.3 ± 1583.2 (n = 47)	0.079

*SpO*_*2*_ hemoglobin oxygen saturation, *SD* standard deviation, *ICU* intensive care unit, *WBC* white blood cells, *RBC* red blood cells, *Hb* hemoglobin, *Ht* hematocrit, *PLT* platelets, *PT* prothrombin time, *INR* international normalized ratio, *APTT* activated partial thromboplastin time, *Fbg* fibrinogen, *CRP* C-reactive protein, *LDH* lactate dehydrogenase, *TP* total protein, *Alb* albumin, *UN* urea nitrogen, *CRE* creatinine, *eGFR* estimated glomerular filtration rate, *UA* uric acid, *Ca* calcium, *IP* inorganic phosphorus, *AST* aspartate aminotransferase, *ALT* alanine aminotransferase, *γ-GTP* gamma-glutamyltransferase, *T-Bil* total bilirubin, *CK* creatine kinase. *Significant difference; one-way analysis of variance for age, RBC, Hb, Ht, and Ca; Kruskal–Wallis test for sex, length of hospital stay, WBC, PLT, PT, PT-INR, APTT, APTT ratio, Fbg, D-dimer, CRP, LDH, TP, Alb, UN, CRE, eGFR, UA, IP, AST, ALT, γ-GTP, T-Bil, and CK.

### Basic profiles and laboratory findings

There was a significant difference in age and lengths of hospital stay among the groups, but there was no significant difference in sex (Table 1). During hospitalization, 16 patients, including three patients classified as mild, died 36.6 ± 25.8 days after admission, and 167 patients were discharged after 13.2 ± 14.1 days. The routine test findings were compared among the four severity groups. Various tests showed significant differences among the groups (Table 1). For example, the levels of WBC, CRP, and lactate dehydrogenase (inflammatory markers) and the activated partial thromboplastin time, fibrinogen, and D-dimer (coagulation-fibrinolysis markers) tended to increase with an increase in disease severity. Regarding renal function markers, there were no significant differences in levels of CRE, inorganic phosphorus, or uric acid among the groups, whereas urea nitrogen (UN) levels and estimated glomerular filtration rate (eGFR) were significantly higher and lower, respectively, in the severe group than those in the other groups. As for liver disorder markers, aspartate aminotransferase, alanine aminotransferase, and gamma-glutamyltransferase levels were higher in the severe group than in the mild group. In contrast, levels of red blood cells, hemoglobin, total protein, albumin, and calcium were lower in the severe group than in the mild group.

### Differences in P-SEP and TM at administration based on disease severity

**Figure 2:**
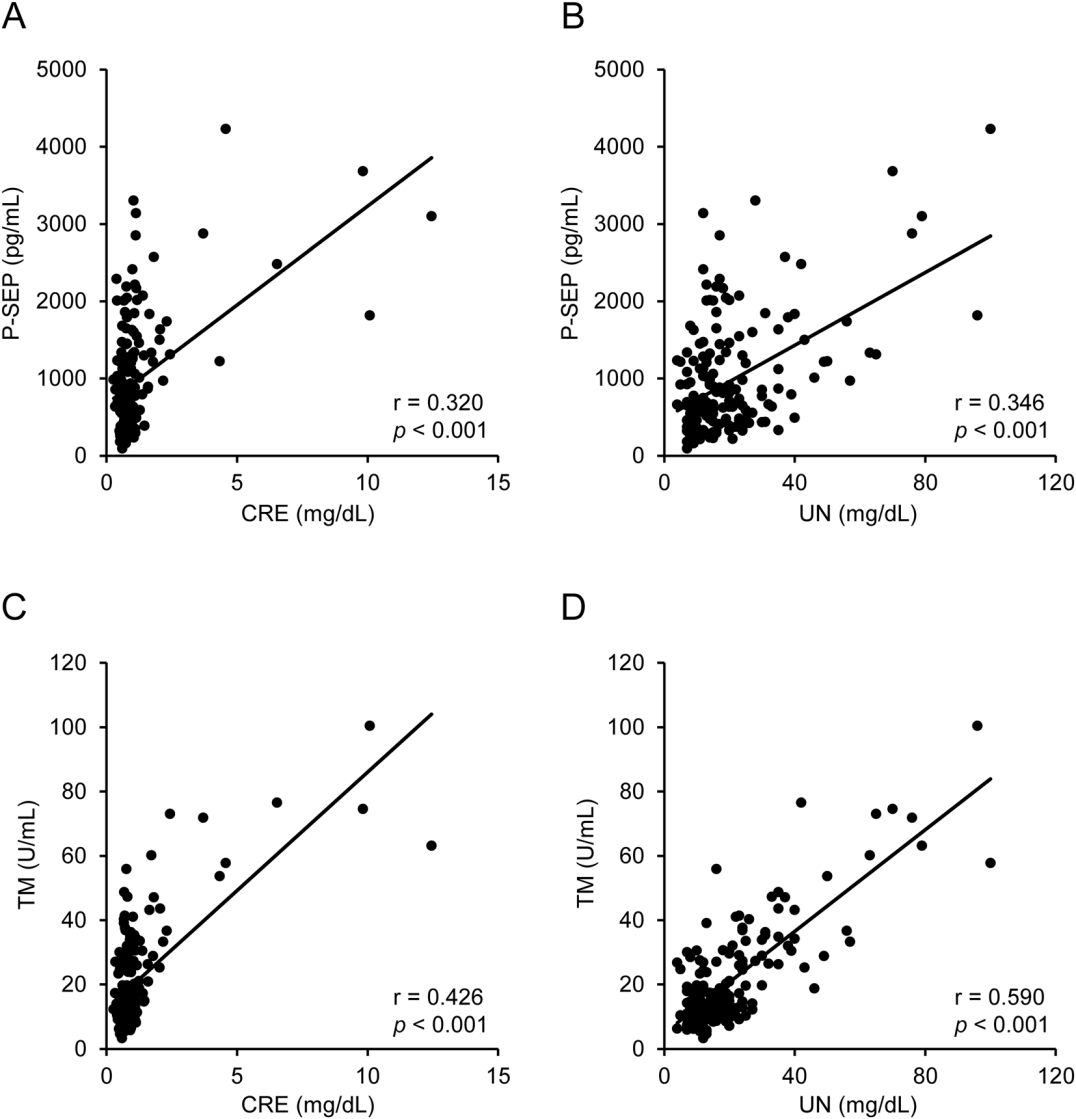
Correlation of presepsin (P-SEP) and thrombomodulin (TM) levels with renal function markers. P-SEP values in all patients were compared with (**A**) creatinine (CRE) and (**B**) urea nitrogen (UN) values (n = 183). The TM values were also compared with the (**C**) CRE and (**D**) UN values (n = 182). One patient who received recombinant human TM treatment was excluded from the TM analysis. Correlations were evaluated using Spearman’s rank correlation test.

Initially, we confirmed whether P-SEP and TM values were associated with levels of the renal dysfunction markers, CRE and UN. P-SEP values were significantly correlated with both CRE and UN values (r = 0.320 and 0.346, respectively, *p* < 0.001) (Fig. 2A,B), although the bias of scatter blots was observed below 3 mg/dL CRE. Similar tendencies were observed in the comparison between TM, CRE, and UN levels. The levels of TM were positively correlated with those of CRE and UN (r = 0.426 and 0.590, respectively, *p* < 0.001) (Fig. 2C,D).

**Figure 3:**
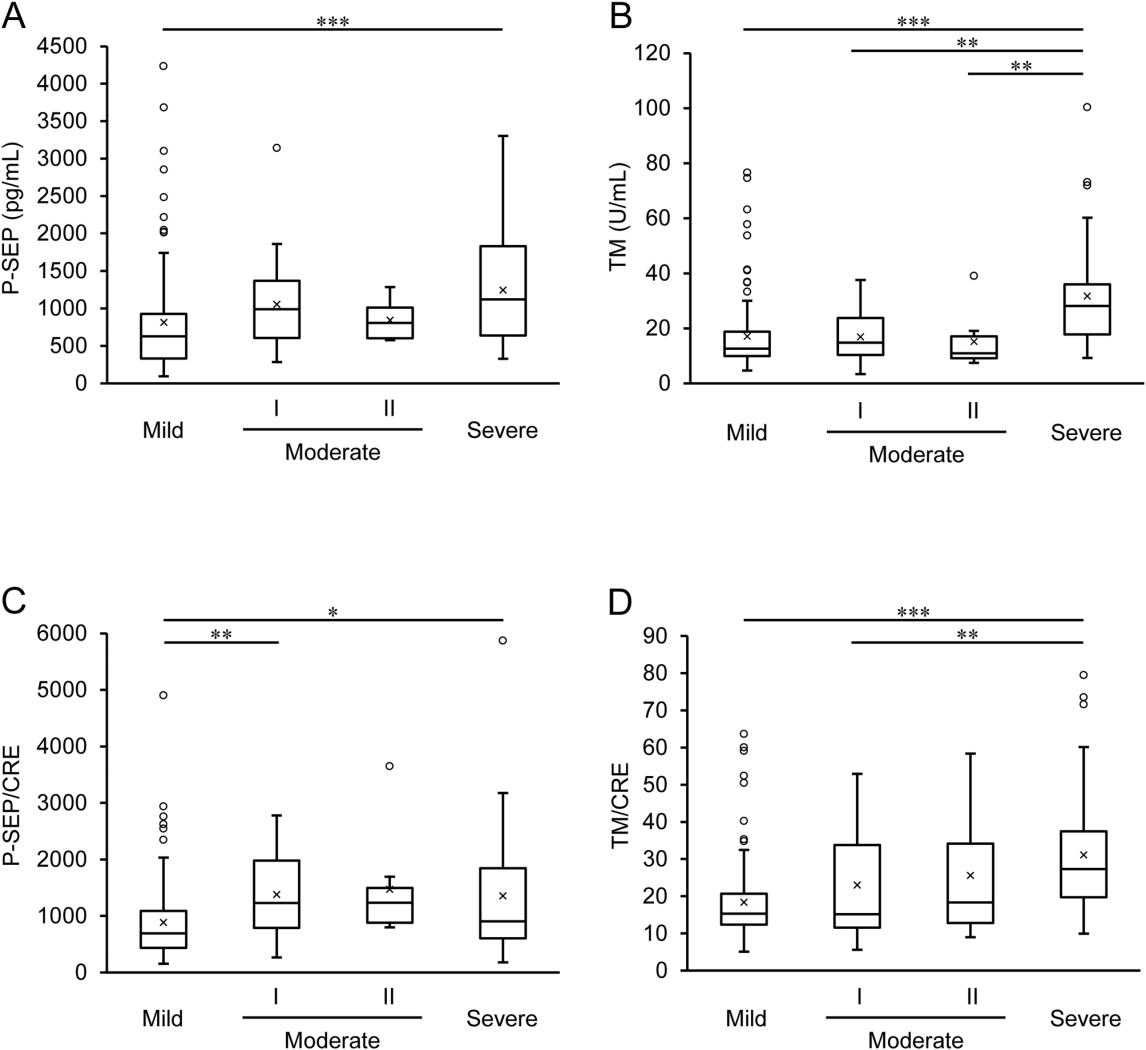
Comparison of presepsin (P-SEP) and thrombomodulin (TM) levels with coronavirus disease (COVID-19) severity. Serum values of (**A**) P-SEP, (**B**) TM, (**C**) P-SEP adjusted for creatinine (CRE) (P-SEP/CRE), and (**D**) TM adjusted for CRE (TM/CRE) in mild (n = 104), moderate I (n = 24), moderate II (n = 8), and severe (n = 47) groups. One patient in the severe group who received recombinant human TM treatment was excluded from the TM analysis. *p < 0.05, **p < 0.01, and ***p < 0.005 indicate the difference between the groups using the Kruskal–Wallis test followed by a post-hoc test with Bonferroni’s correction.

Further, we evaluated P-SEP and TM values in each severity group. The averages of P-SEP values in all the groups were above the cut-off value for the diagnosis of sepsis (> 500 pg/mL). The averages of serum TM values in the mild, moderate I, and moderate II groups were within the reference range (12.1–24.9 U/mL). Using the P-SEP and TM values, we calculated various parameters to determine whether these values would differentiate the patients classified into groups based on their severity (Table 2). P-SEP levels of patients with COVID-19 at administration were significantly higher in the severe group (1245.6 ± 761.5 pg/mL) than in the mild group (812.9 ± 730.7 pg/mL) (*p* < 0.001) (Fig. 3A). TM levels at administration were significantly higher in the severe group (31.7 ± 18.4 U/mL) than the mild group (17.0 ± 13.4 U/mL) (*p* < 0.001), moderate I group (16.9 ± 9.2 U/mL) (*p* = 0.002), and moderate II group (15.2 ± 10.5 U/mL) (*p* = 0.009) (Fig. 3B). There were no significant differences in CRE levels among the groups (Table 1); however, P-SEP and TM were excreted by the kidney, as stated above. Therefore, we adjusted both these values for the CRE value and compared the difference in each value among these groups similarly. Even after adjusting for the CRE value, P-SEP/CRE and TM/CRE were still higher in the severe group than in the mild group (Figs. 3C and 3D). To further confirm whether P-SEP and TM values were changed independently of renal function, multiple linear regression analysis was also performed for severity classification using P-SEP, TM, and CRE values. As a result, the model could explain the severity when even the CRE value was entered in as a variable as shown in the Table 3.

**Table 2:** Data for P-SEP- and TM-related parameters among the severity groups.

	Total	Mild	Moderate I	Moderate II	Severe	*p-*value
P-SEP, pg/mL	957.1 ± 732.7 (n = 183)	812.9 ± 730.7 (n = 104)	1055.3 ± 630.4 (n = 24)	843.4 ± 265.1 (n = 8)	1245.6 ± 761.5 (n = 47)	< 0.001*
TM, U/mL	20.6 ± 15.6 (n = 182)	17.0 ± 13.4 (n = 104)	16.9 ± 9.2 (n = 24)	15.2 ± 10.5 (n = 8)	31.7 ± 18.4 (n = 46)	< 0.001*
P-SEP/CRE	1095.3 ± 855.3 (n = 183)	884.5 ± 705.1 (n = 104)	1377.7 ± 766.3 (n = 24)	1471.2 ± 934.2 (n = 8)	1353.5 ± 1058.3 (n = 47)	< 0.001*
TM/CRE	22.4 ± 14.4 (n = 183)	18.4 ± 11.0 (n = 104)	23.0 ± 15.7 (n = 24)	25.6 ± 17.9 (n = 8)	30.5 ± 16.7 (n = 47)	< 0.001*

*P-SEP* presepsin, *TM* thrombomodulin, *P-SEP/CRE* P-SEP adjusted for CRE, *TM/CRE* TM adjusted for CRE. *Significant difference; Kruskal–Wallis test.

**Table 3:** Multiple linear regression analysis for severity classification (n = 183).

Variables	β	t	*p*-value	95% Confidence interval
P-SEP, pg/mL	0.253	3.442	0.001	0.0002 to 0.0007
TM, U/mL	0.638	7.371	< 0.001	0.039 to 0.067
CRE, mg/dL	− 0.547	− 6.000	< 0.001	− 0.648 to − 0.327

### Comparison between severe survival and non-survival cases

**Figure 4:**
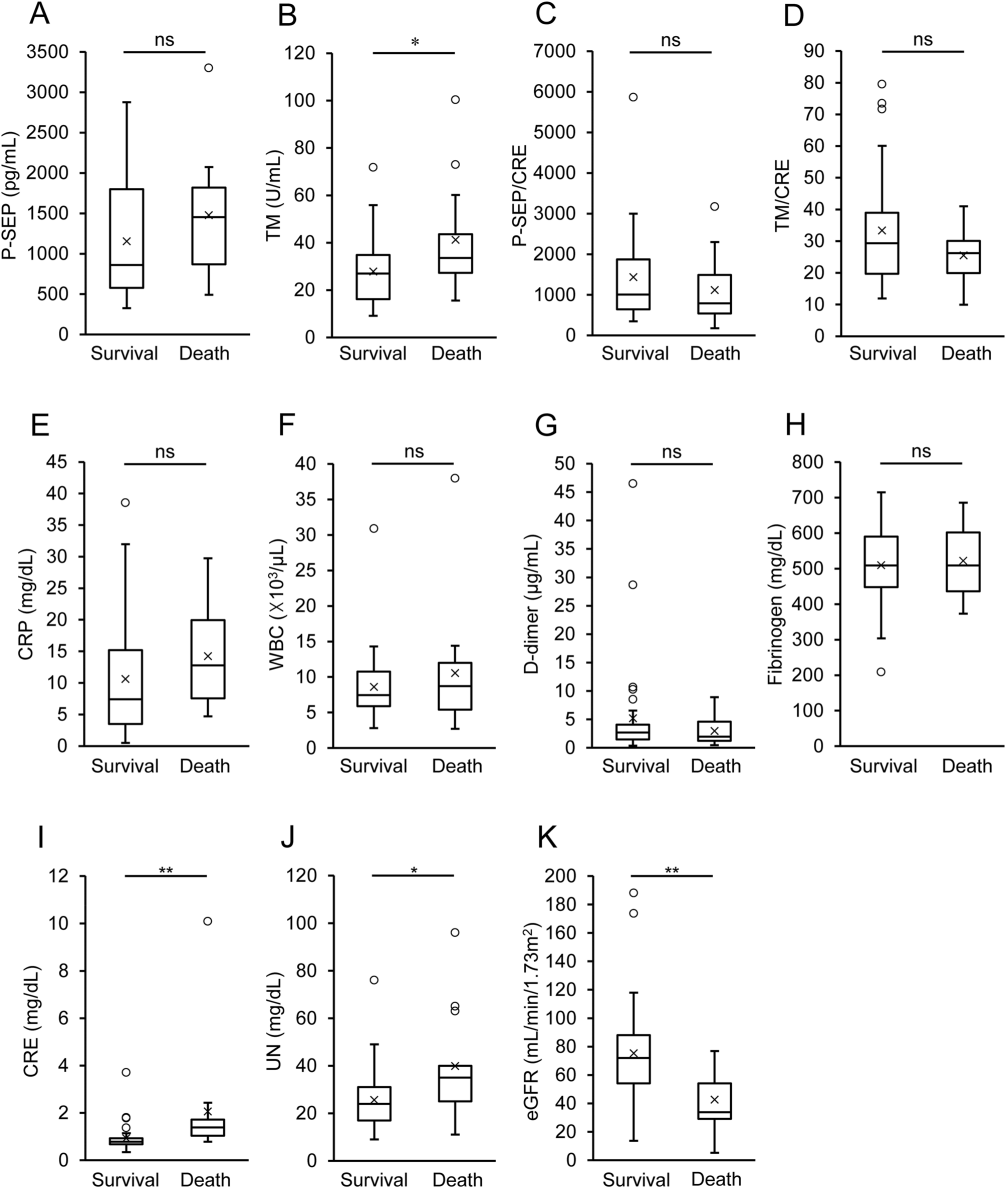
Comparison between severe survivors and non-survivors. Patients in the severe group (n = 47) were further divided into survivors (n = 34) and non-survivors (n = 13). One patient who survived recombinant human thrombomodulin (TM) treatment was excluded from TM analysis. Various markers, (**A**) presepsin (P-SEP), (**B**) TM, (**C**) P-SEP adjusted for creatinine (CRE)(P-SEP/CRE), (**D**) TM adjusted for CRE (TM/CRE), (**E**) C-reactive protein (CRP), (**F**) white blood cells (WBC), (**G**) D-dimer, (**H**) fibrinogen, (**I**) CRE, (**J**) urea nitrogen (UN), and (**K**) estimated glomerular filtration rate (eGFR) were compared between the two cases. *p < 0.05, **p < 0.005; Student’s *t*-test for TM, TM/CRE, fibrinogen, and eGFR; Mann–Whitney *U* test for P-SEP, P-SEP/CRE, CRP, WBC, D-dimer, CRE, and UN; *ns* not significant.

Patients in the severe group were further divided into survivors (n = 34) and non-survivors (n = 13), and the P-SEP and TM levels were compared between them. There was no significant difference in the levels of P-SEP and inflammatory markers (CRP and WBC) between the survivors and non-survivors (Fig. 4). In contrast, non-survivors had significantly higher TM levels (41.23 ± 23.92 U/mL) at admission than the survivors (27.91 ± 14.53 U/mL) (*p* = 0.026), whereas there was no significant difference in the fibrinogen and D-dimer levels between them. When the TM values were adjusted for CRE, the TM/CRE values showed no significant differences between them. As for renal function markers, there was no significant difference in the CRE levels (*p* = 0.141), whereas UN levels were significantly higher in non-survivors than in survivors (*p* = 0.013). Moreover, eGFR was lower in non-survivors than in the survivors (*p* = 0.004).

**Figure 5:**
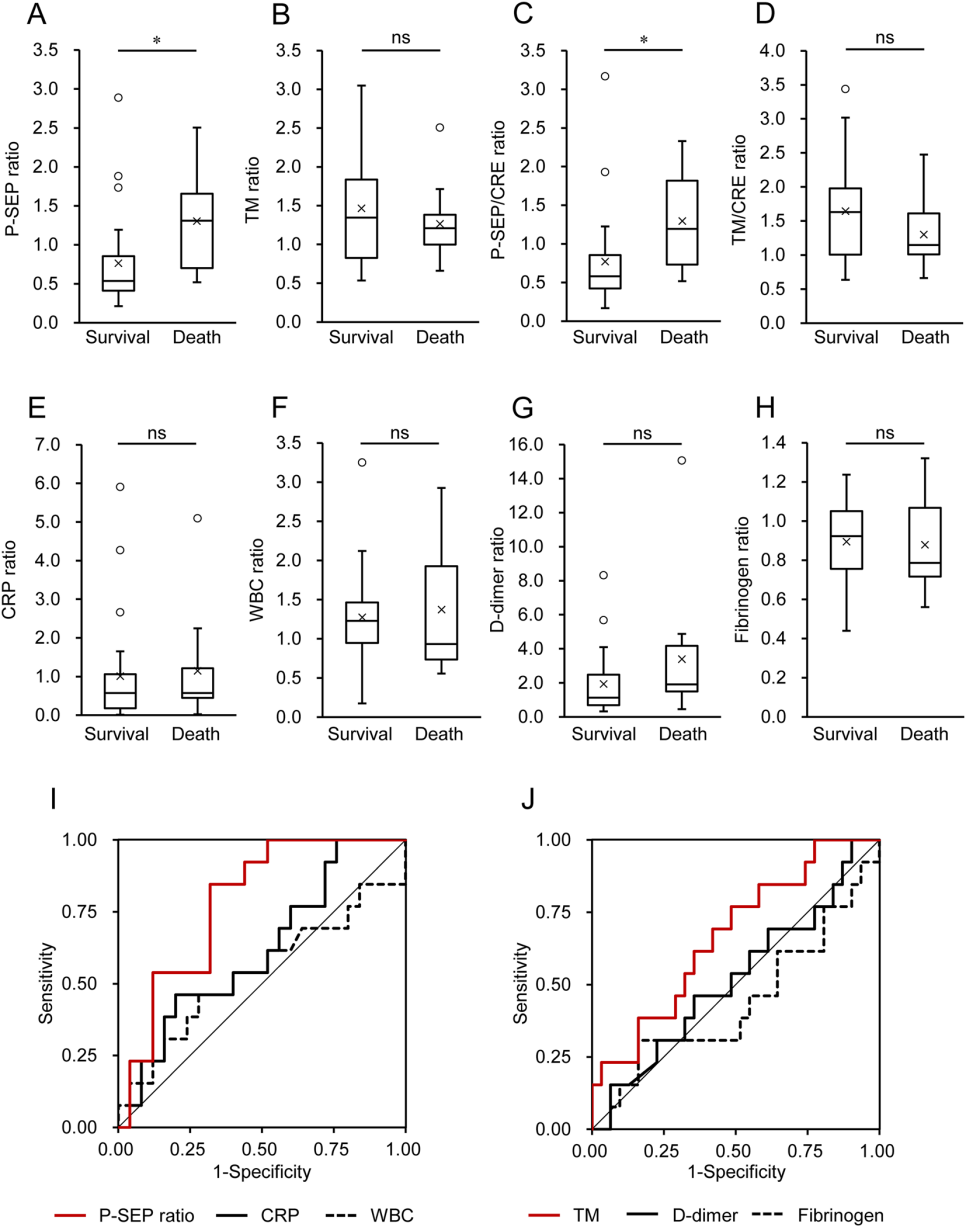
Rate of changes in presepsin (P-SEP) and thrombomodulin (TM), and receiver operating characteristic (ROC) curve analysis. Among the survivors (n = 34) and non-survivors (n = 13), data for those who underwent second P-SEP and TM measurement after admission were further extracted (survivors, n = 21–25, and non-survivors, n = 12–13). One patient who survived recombinant human TM treatment was excluded from TM analysis. The rate change in the levels from the first to the second test for (**A**) P-SEP ratio, (**B**) TM ratio, (**C**) P-SEP/creatinine (CRE) ratio, (**D**) TM/CRE ratio, (**E**) C-reactive protein (CRP) ratio, (**F**) white blood cells (WBC) ratio, (**G**) D-dimer ratio, and (**H**) fibrinogen ratio were compared. *p < 0.005; Student’s *t*-test for TM ratio, TM/CRE ratio, and fibrinogen ratio; Mann–Whitney *U* test for P-SEP ratio, P-SEP/CRE ratio, WBC ratio, CRP ratio, and D-dimer ratio. ROC curve analysis for the rate change in (**I**) P-SEP ratio, CRP, and WBC, and (**J**) TM, D-dimer, and fibrinogen for predicting in-hospital mortality in the severe group; *ns* not significant.

Furthermore, we focused on the changes in P-SEP and TM levels after administration. Survivors (n = 25 for P-SEP and n = 24 for TM) and non-survivors (n = 13) with more than two P-SEP and TM measurements were analyzed. The elapsed number of days (mean ± standard deviation (SD)) between the first and second tests for both P-SEP and TM was 4.1 ± 2.2 days. As for P-SEP, when the ratio of the second measurement value to the first value (P-SEP ratio) was calculated, there was a significant difference between survivors and non-survivors (*p* = 0.004) (Fig. 5A). The ratio in the survivors was less than 1.0 (0.77 ± 0.61), while the ratio in non-survivors was elevated (1.30 ± 0.70). Moreover, when P-SEP was adjusted for CRE, the change in P-SEP/CRE (P-SEP/CRE ratio) was still higher in non-survivors (*p* = 0.002) (Fig. 5C). In contrast, TM values in both cases increased from the first measurement at admission, and the TM ratio in survivors and non-survivors was 1.46 ± 0.72 and 1.26 ± 0.46, respectively, while there was no significant difference in the ratios between both groups (Fig. 5B). Similarly, there was no significant difference in ratios of CRP, WBC, D-dimer, and fibrinogen between the survivors and non-survivors (Fig. 5E–H).

### Receiver operating characteristic (ROC) curve analysis

We further evaluated the ability of P-SEP and TM values to predict in-hospital mortality using ROC curves (Fig. 5). Since the P-SEP ratio showed stronger statistical power than that of the P-SEP value in terms of differentiation between survivors and non-survivors, the P-SEP ratio was used for ROC analysis. The areas under the ROC curves (AUROC) (95% confidence interval (CI), asymptotic significance) of models using the P-SEP ratio, CRP, and WBC were 0.782 (95% CI 0.637–0.927, *p* = 0.005), 0.615 (95% CI 0.428–0.803, *p* = 0.249), and 0.537 (95% CI 0.327–0.747, *p* = 0.712), respectively (Fig. 5I). When ROC analysis was performed using TM, D-dimer, and fibrinogen, TM showed an insignificantly higher AUROC than that of the other coagulation-fibrinolysis markers (Fig. 5J). The AUROC of TM, D-dimer, and fibrinogen were 0.667 (95% CI 0.496–0.839, *p* = 0.083), 0.439 (95% CI 0.240–0.639, *p* = 0.529), and 0.520 (95% CI 0.329–0.710, *p* = 0.837), respectively. According to the largest Youden Index, the optimum cut-off values for the P-SEP ratio and TM were 0.67 and 27.3 U/mL, respectively. With regard to kidney disorder markers, CRE and UN showed good performance, and their AUROC were 0.804 (95% CI 0.670–0.937, *p* < 0.001) and 0.820 (95% CI 0.684–0.955, *p* < 0.001), respectively (data not shown).

### Daily changes in P-SEP and TM levels

**Figure 6:**
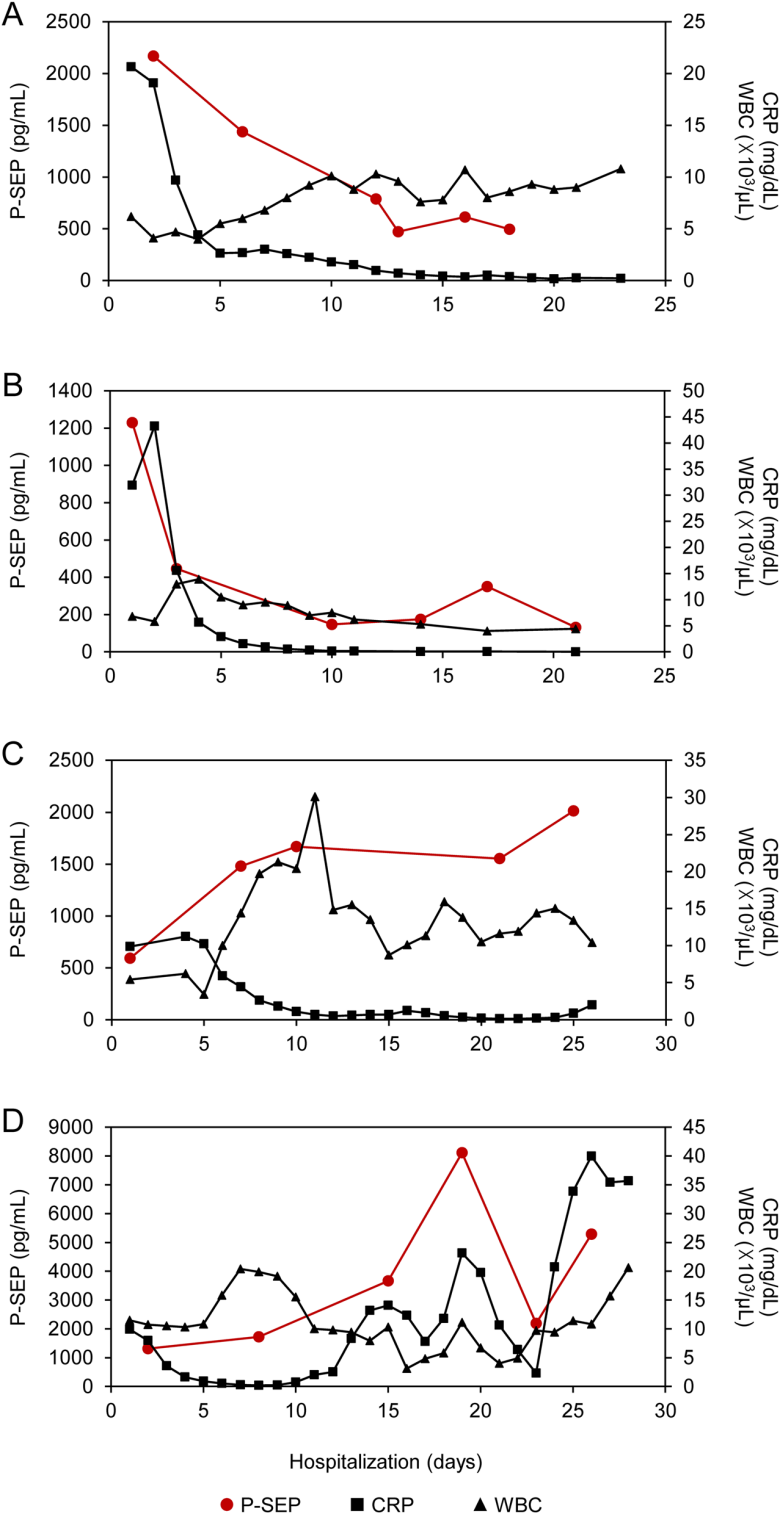
Trend for presepsin (P-SEP) levels during hospitalization in survivors and non-survivors. Two representative graphs of (**A**,**B**) survivors and (**C**,**D**) non-survivors showing typical changes of four points or more in P-SEP and other inflammatory markers (C-reactive protein (CRP) and white blood cells (WBC)) during hospitalization.

**Figure 7:**
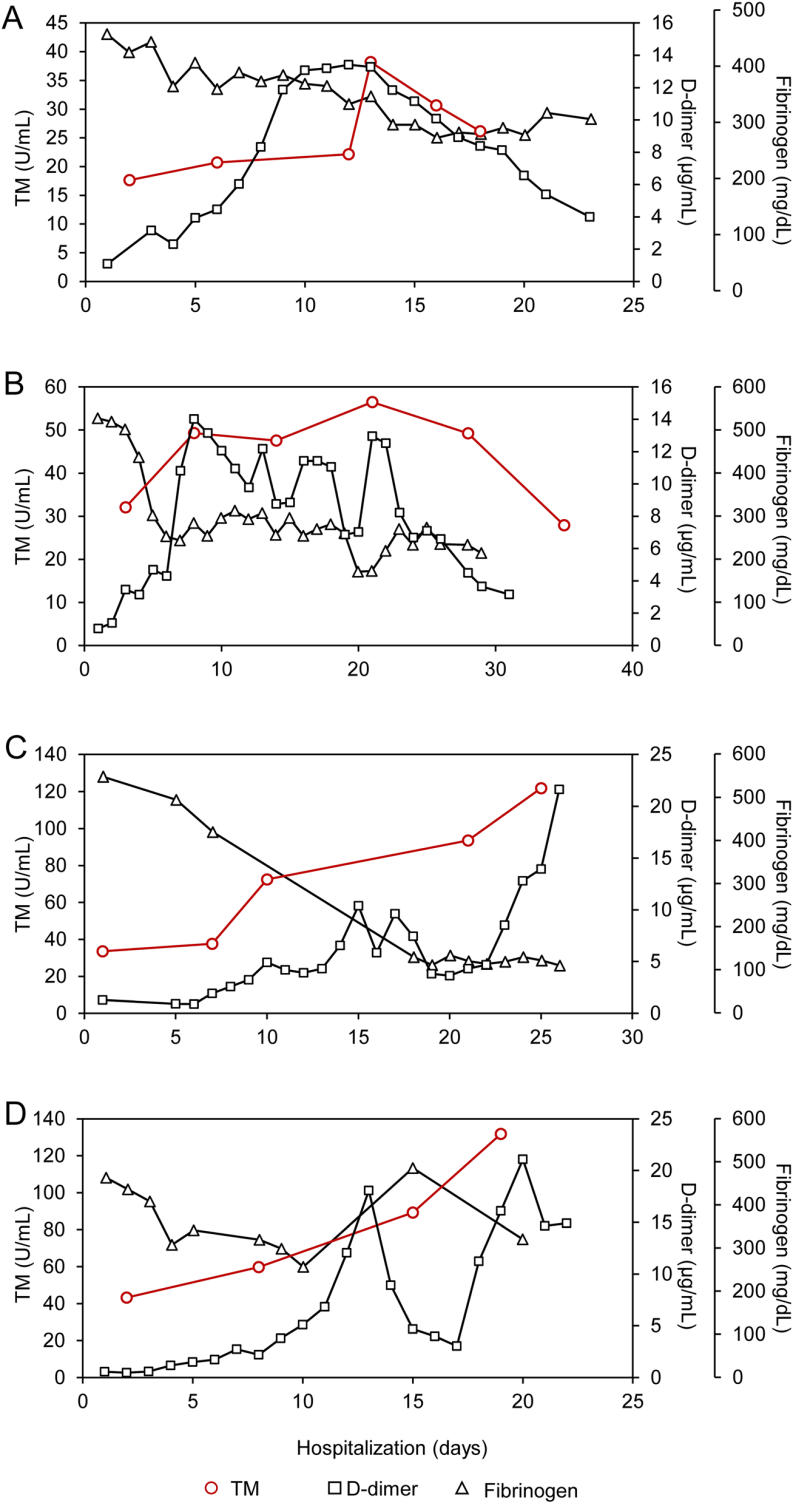
Trend for thrombomodulin (TM) levels during hospitalization in survivors and non-survivors. Two representative graphs of (**A**,**B**) survivors and (**C**,**D**) non-survivors showing typical changes of four points or more in TM and other coagulation-fibrinolysis markers (D-dimer and fibrinogen) during hospitalization.

Since the change in P-SEP concentration can be useful for predicting mortality, we monitored P-SEP and TM levels in patients in the severe group. The representative time courses of P-SEP and TM for survivors and non-survivors are shown in Fig. 6 (for P-SEP) and Fig. 7 (for TM). As shown in Fig. 5, there was no significant difference in P-SEP levels on admission between the survivors and non-survivors. However, in survivors, the levels of P-SEP on second testing decreased and continued to decline or remained low until the discharge date (Fig. 6A,B). Similar tendencies were observed for CRP and WBC values. In contrast, in non-survivors, P-SEP values did not decrease on the second testing day and drastically increased before death (Fig. 6C,D). As for TM, as shown in Fig. 5, the values on admission were higher in non-survivors than those in the survivors (Fig. 7). However, unlike P-SEP, a decrease in TM levels was not observed in survivors (Fig. 7A,B), even though fibrinogen levels tended to decrease until discharge. Interestingly, D-dimer levels increased until the middle of hospitalization and then decreased to normal levels. In contrast, TM and D-dimer levels increased drastically, similar to P-SEP among non-survivors, while an increase in fibrinogen levels was not observed (Fig. 7C,D).

## Discussion

Sepsis, respiratory failure, and ARDS are among the most common symptoms observed in COVID-19 non-survivors^30^; thus, in this study, we focused on the sepsis biomarker, P-SEP, and endothelial dysfunction observed in ARDS, which are still unclear topics in the literature. For comparison with P-SEP and TM, we selected CRP and WBC as representative inflammatory biomarkers, and fibrinogen and D-dimer as representative coagulation-fibrinolysis biomarkers. These are common testing measures upon patient admission, and we thought that they could be compared during inflammatory conditions such as sepsis and endothelial dysfunction. Moreover, to our knowledge, no study has utilized CRE adjustment for evaluating patients with COVID-19 or evaluated the change in these values between two time points. With regard to the classification of severity, P-SEP and TM levels reflected the aggravation of symptoms, while other major markers, such as WBC, CRP, fibrinogen, and D-dimer, were also differentiated based on severity. In contrast, only TM levels showed a significant difference in mortality prediction.

TM plays a role in the suppression of the coagulation pathway by activating protein C and inactivating activated coagulation factors V and VIII in cooperation with thrombin^31,32^. However, under various pathological conditions, the expression of TM can be modulated. Cytokines are major factors in the onset of disseminated intravascular coagulation associated with severe infectious diseases such as sepsis. Many tissue factors are produced from monocytes, macrophages, and vascular endothelial cells by inflammatory cytokines such as lipopolysaccharide, tumor necrosis factor, and interleukin-1, resulting in remarkable coagulation activation^33^. The released lipopolysaccharide and cytokines suppress the expression of TM, an anticoagulant protein in vascular endothelial cells, and accelerate coagulation activation. In addition, lipopolysaccharide and cytokines overexpress plasminogen activator inhibitor, which is a fibrinolytic inhibitor, in vascular endothelial cells and suppress fibrinolysis, resulting in multiple organ failure due to residual microthrombus^34,35^. Thus, suppression of TM expression in endothelial cells is associated with the pathological progression of coagulation. At the same time, TM is released from the injured cells into circulation, and the elevated plasma TM levels can be used as a biomarker for endothelial cell injury. With regard to COVID-19, the expression of TM in patient’s injured pulmonary endothelial cells is also decreased and is associated with immune cell infiltration in the lungs^36^. Moreover, high circulating platelet aggregates were observed in non-survivor patients with COVID-19 and correlated with TM levels more than with D-dimer and fibrinogen levels^37^, as observed in our current study. Although contradictory results for blood TM levels as a biomarker have been reported, high TM levels may reflect endothelial disorder and coagulation promotion, which could prove fatal, similar to the present study findings.

Furthermore, P-SEP levels did not show a significant difference between survivors and non-survivors, whereas the change in P-SEP values reflected mortality. P-SEP is a relatively novel septic marker, and many studies have demonstrated its usefulness for the early diagnosis of sepsis and prognosis compared with other septic markers. The AUC of P-SEP calculated from the ROC curves in the infection group was significantly greater than that of procalcitonin, CRP, and IL-6^8^. P-SEP is more useful in classifying the stage of sepsis than other septic markers (procalcitonin, CRP, and IL-6)^38^. Considering that most non-survivors with COVID-19 showed symptoms of sepsis^1^, the dynamics of P-SEP values in patients with COVID-19 might be similar to those in patients with sepsis. In this study, 7 patients in the non-survivor group died due to sepsis. However, when serum samples were collected at admission for P-SEP and TM levels assessment, sepsis was deniable, at least, in cases of the 4 patients, while 1 patient had already developed sepsis at admission. Generally, P-SEP values do not increase in patients with viral infections; however, SARS-CoV-2 can directly infect monocytes to reduce CD14^+^/CD16^-^ classical monocytes and increase CD14^+^/CD16^+^ intermediate monocytes^39^, which have an increased phagocytic function^40^, resulting in the release of cytokines including P-SEP in the early stage of the disease. This could explain the high P-SEP values even in the mild group. To our knowledge, this is the first study to evaluate P-SEP values at multiple time points as well as the change in values after admission for patients with COVID-19, except for the case studies. In a previous study that investigated P-SEP levels in patients with sepsis, the P-SEP levels gradually increased from the early stage based on the severity of sepsis^8,41^. Elevation in P-SEP values in non-survivors was observed over time; however, these elevations were not observed in survivors^42^. A recent study has reported that P-SEP is one of the valuable prognostic biomarkers in assessing ICU mortality risk in COVID-19 patients independent of dexamethasone administration^43^. As with sepsis, changes in P-SEP levels might provide more important information for the prediction of mortality, even in COVID-19 patients. In addition, some non-survivor patients with COVID-19 showed renal dysfunction, and in this study, some patients in the severe group (particularly the non-survivor group) had high CRE levels. However, significant differences in P-SEP and TM values remained even after CRE adjustment, which indicated that both might be used as predictive markers, apart from renal function. Moreover, 10 out of 183 patients underwent the renal replacement therapy (RRT)(continuous hemodialysis or continuous hemodialysis and filtration). Among the 10 patients, 9 patients were classified in the severe group: 8 patients in the non-survivors and 1 patient in the survivors, while there were no relationships of P-SEP and TM levels between RRT-treated and non-treated patients.

One of the limitations of this study was its small total sample size which limited the power of analysis, especially, the difference in the number of groups based on severity, as the severity was classified after measuring P-SEP and TM; for example, the number of moderate I and II patients was small that might lead to result bias. In addition, there were relatively many severely ill patients because many patients were transferred from other hospitals after becoming severely ill. For the transferred patients, data at the time of onset were not available. Therefore, this study was based on the results obtained at the time of admission to our hospital, and it is difficult to evaluate the condition at the time of onset from these results. However, the changes in P-SEP and TM measurements from the point of admission are expected to provide important information regarding the severity of COVID-19.

In conclusion, TM measurement and continuous P-SEP measurement, possible independent of renal function markers, would be useful in predicting mortality in patients with COVID-19.

## Methods

### Samples

Consecutive patients admitted to the Tokyo Medical and Dental University Hospital between April 7, 2020, and December 14, 2020 were analyzed. We retrospectively enrolled the patients diagnosed with COVID-19 by detecting SARS-CoV-2 RNA using real-time polymerase chain reaction according to the inclusion criteria (see Fig. 1): patients were greater than 18 years of age, SpO_2_ data on admission were obtained before oxygen therapy, and serum samples were collected within 5 days of admission with reference to the previous reports^11,27,42,44,45^. Regarding to the TM analysis, one patient who received human recombinant TM (rhTM) treatment was excluded. The samples were residual sera collected between April 10, 2020, and December 15, 2020 for medical use from patients with COVID-19 by the infection control department and had been stored at − 80 °C.

All methods were performed in accordance with the relevant guidelines and regulations. Written informed consent was obtained from all the patients. This study was approved by the ethics committee of the Faculty of Medicine, Tokyo Medical and Dental University (No. M2020-110).

### Measurement of P-SEP and TM

The stored patient serum samples were thawed in a refrigerator at 4 °C for 24 h. Serum P-SEP and TM levels were measured using commercially available assay kits based on a chemiluminescent enzyme immunoassay: STACIA CLEIA Presepsin (normal range: 55–184 pg/mL^46^) (LSI Medience Corporation, Tokyo, Japan) and STACIA CLEIA TM (normal range: 12.1–24.9 U/mL^47^) (LSI Medience Corporation, Tokyo, Japan), respectively. These measurements were performed using the STACIA clinical assay system (LSI Medience Corporation, Tokyo, Japan).

### Data collection

The medical history of patients, including clinical laboratory data, SpO_2_, transfer to the ICU, use of ventilator support, and the administration of rhTM, was extracted from electronic medical records.

### Statistical analyses

The results are expressed as the mean ± SD. All data were statistically analyzed using SPSS version 25.0 (IBM Corp., Armonk, NY, USA). The Kolmogorov–Smirnov test was performed to assess whether the data were from a Gaussian distribution, and nonparametric tests were used to evaluate the non-normal data. Correlation analyses were performed using Spearman’s rank correlation test (Fig. 2). The one-way analysis of variance or Kruskal–Wallis test, followed by a post-hoc test with Bonferroni’s correction, was used to compare variations in the levels of P-SEP, TM, and major biochemical parameters with the severity of COVID-19 (Tables 1, 2, and Fig. 3). Multiple regression analysis was performed through enter method and stepwise process to evaluate P-SEP and TM values as independent biomarkers (Table 3). Differences in changes in P-SEP, TM, and the other biomarker levels between survival and non-survival cases were evaluated using an unpaired Student’s *t*-test or Mann–Whitney *U* test (Figs. 4 and 5). Statistical significance was set at *p* < 0.05. The ROC curve and the corresponding AUROC were used to assess and compare the prognostic prediction using P-SEP and other biomarkers (Fig. 5).

## Data Availability

The datasets used and analyzed during the current study are available from the corresponding authors upon reasonable request.
